# From Bench to Bladder: The Rise in Immune Checkpoint Inhibition in the Treatment of Non-Muscle Invasive Bladder Cancer

**DOI:** 10.3390/cancers17071135

**Published:** 2025-03-28

**Authors:** Caitlin P. Burns, Jacob M. Parker, Dylan M. Schaap, Mark R. Wakefield, Yujiang Fang

**Affiliations:** 1 Department of Microbiology, Immunology & Pathology, Des Moines University College of Osteopathic Medicine, West Des Moines, IA 50266, USA; caitlin.p.burns@dmu.edu (C.P.B.); jacob.parker@dmu.edu (J.M.P.); dylan.m.schaap@dmu.edu (D.M.S.); 2 Department of Surgery, University of Missouri School of Medicine, Columbia, MO 65212, USA; wakefieldmr@health.missouri.edu; 3 Ellis Fischel Cancer Center, University of Missouri School of Medicine, Columbia, MO 65212, USA

**Keywords:** immunotherapy, tumor microenvironment, anti-PD-1/PD-L1, anti-CTLA-4, immune checkpoint inhibitors

## Abstract

Non-muscle invasive bladder cancer (NMIBC) represents a significant clinical challenge due to its high recurrence rate and need for frequent monitoring. The current treatment modality is bacillus Calmette–Guérin (BCG) therapy combined with chemotherapy after transurethral resection of the bladder tumor (TURBT), which is highly effective in most patients. Yet, the cancer becomes resistant to these treatments in 30–40% of patients, necessitating the need for new treatment modalities. In the cancer world, the development of immune checkpoint inhibitors that target molecules, such as programmed cell death protein-1 (PD-1), its ligand, PD-L1, and Cytotoxic T-lymphocyte-associated protein-4 (CTLA-4), have revolutionized the treatment of many cancer types. PD-1/PD-L1 and CTLA-4 are shown to be upregulated in NMIBC in certain circumstances. PD-1/PD-L1 interactions play a role in immune evasion by suppressing T cell activity within the tumor microenvironment (TME), while the binding of CTLA-4 on T cells leads to downregulation of the immune response, making these pathways potential immunotherapeutic targets in NMIBC. This review seeks to understand the role of these therapies in treating NMIBC. We explore the cellular and non-cellular immune landscape in the TME of NMIBC, including Tregs, T effector cells, macrophages, B cells, and relevant cytokines. We also discuss the biological role of PD-1/PD-L1 and CTLA-4 while covering the rationale for these immunotherapies in NMIBC. Finally, we cover key clinical trials that have studied these treatments in NMIBC clinically. Such a study will be helpful for urologists and oncologists to manage patients with NMIBC more effectively.

## Simple Summary

Non-muscle invasive bladder cancer (NMIBC) poses a significant challenge due to its high recurrence rate and resistance to treatments, such as BCG therapy, in many patients. Due to the need for new treatment methods, this review aims to explore the role of immune checkpoint inhibitors (ICI) like PD-1/PD-L1 and CTLA in treating NMIBC. These inhibitors are known to help the immune system fight cancer more effectively. The findings could provide urologists and oncologists with better tools to manage NMIBC, potentially leading to more effective treatments and improved patient outcomes.

## 1. Introduction

Urinary bladder cancer (UBC) is a prevalent form of cancer and the most common malignancy of the urinary tract, with an estimated 83,190 new cases in the United States each year [1]. Non-muscle invasive bladder cancer (NMIBC) is a heterogeneous UBC classification that accounts for approximately 75% of UBC diagnoses and is over four times more common in men than women [2,3]. UBC predominantly affects older adults, with the average age of diagnosis being 73, and 90% of diagnoses occurring in individuals aged 55 or older [3,4]. Among NMIBC diagnoses, 60–70% are confined to the bladder mucosa (Ta), 20–30% invade subepithelial connective tissue (T1), and 10% are carcinomas in situ (CIS) [5,6]. The highest risk factors for developing UBC are age, sex, genetic susceptibility, and tobacco smoking [7]. First-degree relatives of UBC patients have a two-fold higher risk of developing UBC, and tobacco smoking accounts for 50% of diagnoses [7,8]. Furthermore, NMIBC patients are divided into low, intermediate, high, and very high-risk groups with 0.93%, 4.9%, 9.6%, and 40% probabilities of progression, respectively. These classifications are based on tumor type, number, diameter, patient age, and presence of CIS [9].

Approximately half of untreated NMIBC patients progress to muscle-invasive bladder cancer (MIBC), and even after treatment recurrence, rates of NMIBC are 70–80% [4,10]. Current treatment options for NMIBC include transurethral resection of bladder tumor (TURBT), intravesical bacillus Calmette–Guérin (BCG) immunotherapy, adjuvant intravesical treatment, and intravesical chemotherapy [11]. Despite being one of the most effective therapies following TURBT, approximately 30–40% of NMIBC patients are unresponsive to BCG therapy and experience tumor recurrence or progression [12]. BCG also has adverse effects including cystitis, fever, fatigue, and rarely, sepsis [13,14,15]. Intravesical chemotherapies are offered as an alternative to BCG immunotherapy, and the most common treatments are mitomycin C, epirubicin, and doxorubicin [16]. Mitomycin C is used for low- and intermediate-risk NMIBC patients following TURBT and, when maintained, has one-, two-, and five year-recurrence-free survival (RFS) rates of 84%, 75%, and 51%, respectively [17]. However, there are no clinical guidelines on optimal schedule or dosing. Epirubicin is less effective than BCG in preventing disease progression and patients often experience negative side effects such as cystitis, dysuria, pollakiuria, and bladder irritation [18]. Doxorubicin following TURBT is associated with lower risks of recurrence but does not affect time to progression [19]. Given the current challenges and limitations in treating NMIBC, new therapeutic strategies are necessary to improve patient outcomes and comfort.

One potential strategy that is being investigated is targeting immune checkpoint pathways. Immune checkpoints refer to the co-stimulatory and inhibitory signals expressed between immune cells that regulate T cells and normally enable self-tolerance [20]. Tumor cells often exploit these pathways by overexpressing inhibitory ligands, preventing the T cells (particularly CD8^+^ T cells) from attacking them. A notable immunosuppressive checkpoint receptor is the programmed cell death protein-1 (PD-1), expressed on the surface of many immune cells, including activated T cells. Its ligand, PD-L1, is expressed on tumor cells and, upon interaction with PD-1, suppresses CD8^+^ T cell cytotoxicity and proliferation [20,21]. In bladder cancer, high expression of PD-L1 has been linked to higher chances of metastasis and shortened survival lengths [22].

Cytotoxic T-lymphocyte-associated protein 4 (CTLA-4) is a protein expressed by T cells that competes with CD28 for binding to CD80 (B7.1) or CD86 (B7.2) expressed on the surface of antigen-presenting cells (APC). Binding of CD28 typically activates T cells, whereas CTLA-4 leads to an inhibitory response [20,23] and downregulation of the immune response. Due to their protective effect on tumor cells, immune checkpoint inhibitors (ICI) of PD-1, PD-L1, and CLTA-4 have emerged as promising immunotherapies in the treatment of NMIBC. This review will discuss the pathophysiology of NMIBC, highlighting the major factors influencing tumor progression within its microenvironment, the role of PD-L1 and CTLA-4 in UBC, and current clinical trials targeting these checkpoints.

## 2. Pathophysiology of NMIBC and the Tumor Microenvironment (TME)

### 2.1. Molecular and Histological Characteristics of NMIBC

To diagnose NMIBC, a biopsy is performed to examine tissue changes caused by genetic and epigenetic alterations. Key molecular alterations in NMIBC include fibroblast growth factor-3 (FGFR3), phosphatidylinositol 3-kinase (PIK3CA), and TP53 mutations, as well as chromosomal alterations and DNA methylation [24,25]. These mutations may also be predictive markers of NMIBC relapse [26].

FGFR3 mutations are present in approximately 70% of NMIBC cases compared to about 15% of MIBC cases [27,28]. These mutations promote cell proliferation, survival, and angiogenesis and are often associated with lower-grade papillary tumors [27]. FGFR3 is a tyrosine kinase receptor that controls the growth and activation of fibroblasts. Upon activation, FGFR3 activates the Ras/MAPK pathway and potentially the PI3K/AKT pathway [27,29,30,31,32,33,34,35,36]. PIK3CA mutations occur in 26% of NMIBC cases, and while there is less literature describing these mutations compared to FGFR3, we know that when both PIK3CA and FGFR3 mutations are present, this is a risk factor for relapse [26,37]. Additionally, patients who exhibit TP53, PIK3CA, and ataxia telangiectasia-mutated (ATM) mutations are shown to respond better to ICIs, which may be a crucial factor to consider when analyzing a patient’s tumor profile [38]. TP53 mutations are associated with more aggressive phenotypes appearing in 48% of T2 NMIBC patients, leading to the inactivation of the p53 tumor suppressor. This is associated with high levels of recurrence and progression to MIBC [39,40]. In normal cells, p53 can become activated by the ATM/ataxia-telangiectasia Rad3-related (ATR) pathway, which occurs when DNA is damaged. In NMIBC, a truncated p53 is created, which leads to loss of function and increased cell proliferation [41].

**Figure 1:**
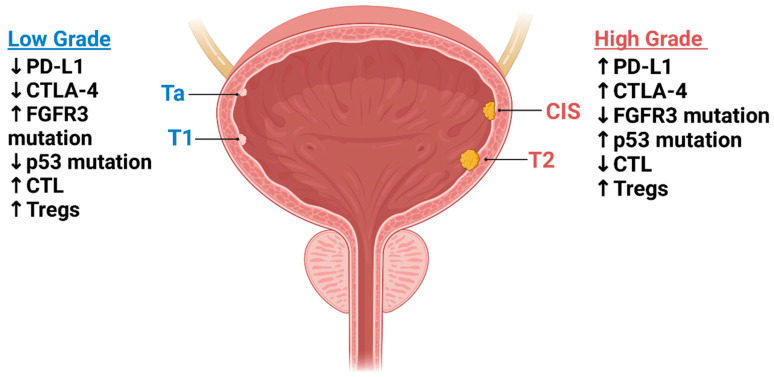
Staging and markers upregulated (↑) or downregulated (↓) in low-grade vs. high-grade NMIBC. Ta, T1, and CIS denote NMIBC, while T2 is considered MIBC. The grading of these tumors is based on how far they have invaded the muscle layer of the bladder.

Chromosomal aberrations are common in NMIBC; complete loss of chromosome nine is found in 53% of NMIBC cases, leading to a lack of inhibition in the cell cycle [42,43]. Alterations in 8q lead to cellular dysregulation of extracellular matrix (ECM) synthesis, transform growth factor-β (TGF-β) pathways, and cause uncontrolled cell division and tumor progression. Genetic gains of chromosomes are common and can cause immortalization of cancer cells, as seen with chromosome 5p [42,44,45]. Many of these alterations contribute to tumor heterogeneity and are different in every patient. Trends can be seen between high-grade and low-grade tumors and other molecular markers tend to be upregulated in these individuals (Figure 1).

This section highlights key molecular changes that occur in NMIBC cells that result in cellular and extracellular changes that can be observed histologically. Mutations that cause increased cell proliferation will result in visible hyperplasia, which will help grade and stage the cancer. The staging and grading rules are highlighted elsewhere in this paper.

### 2.2. Histological Grading and Staging

The 2022 World Health Organization (WHO) classification categorizes NMIBC into two main categories: papillary urothelial neoplasms and flat urothelial lesions. Papillary urothelial neoplasms begin as a papilloma, which is a benign tumor with a fibrovascular core lined by normal appearing urothelium. It is rare for a papilloma to recur after complete removal, but if not treated or completely removed the papilloma will progress to a papillary urothelial neoplasm of low malignant potential. These cells show significant nuclear enlargement, pleomorphism, hyperchromatic, and frequent mitoses. These histologic characteristics have a higher risk of recurrency and progression to MIBC. Finally, there is high-grade papillary urothelial carcinoma, which is more aggressive with significant atypia, pleomorphism, and frequent mitoses. This is associated with poor prognosis and a higher risk of progression to muscle-invasive bladder cancer [46,47].

Flat urothelial lesions are primarily characterized by reactive urothelial atypia, which consists of benign changes in the urothelium in response to inflammation or irritation and has no architectural or cytologic atypia association. Flat urothelial lesions can progress and exhibit urothelial dysplasia, which consists of moderate nuclear atypia but lacks the full thickness to meet a CIS. Dysplasia may regress, remain stable, or progress to CIS. High-grade flat lesions involving the entire thickness of the urothelium are the diagnosis of CIS. These cells show severe nuclear atypia, pleomorphism, and frequent mitosis. This diagnosis carries a high risk of progression to muscle-invasive disease [47].

The grading process of cancer involves cytologic and architectural atypia and is named low-grade or high-grade based on severity. Where grading is based on physical changes in the cells, the staging of NMIBC is classified based on the depth of cancer invasion in the bladder wall. The stages include Ta, T1, or CIS, where Ta is the least invasive and CIS is the most invasive [47].

### 2.3. The Role of the TME in NMIBC Progression

The TME refers to the complex ecosystem within and surrounding the tumor, consisting of intratumoral cancer cells, stromal cells, endothelial cells, and immune cells (T cells, B cells, macrophages, natural killer (NK) cells, dendritic cells (DC), myeloid-derived suppressor cells (MDSCs), and stromal cells) and non-cellular components (ECM, cytokines, chemokines, growth factors, and metabolic byproducts). The composition of the TME can either support or suppress tumor growth and impact tumor response to treatment [48].

NMIBC is confined to the inner layer of the bladder, resulting in a more confined TME than MIBC [48]. Despite this, the TME may still facilitate a more aggressive NMIBC phenotype. For example, high-grade NMIBC often has reduced inflammation or “colder” tumors [49], characterized by low immune cell infiltration and lack of an effective immune response leading to the secretion of cytokines and chemokines that inhibit T cell recruitment and activation [50]. These cold tumors exhibit lower density and distribution of CD8^+^ T cells, lower B7/PD-L1 expression, and decreased major histocompatibility complex (MHC) class I expression on tumor cells, resulting in a diminished immune response. The tumor-associated macrophages (TAMs) then promote immunosuppression by attracting regulatory T cells (Tregs) via CCL20 [50]. Rapid cell growth within aggressive NMIBC may lead to hypoxia, resulting in a shift to anaerobic metabolism and increased lactate, further inhibiting T cell function [51].

Cancer-associated fibroblasts also play a key role in facilitating NMIBC progression within the TME. TGF-β, commonly secreted in colder tumors, drives fibroblast activation, immune suppression, ECM remodeling, and myofibroblast differentiation. Activated fibroblasts and myofibroblasts secrete collagen and fibronectin, increasing tissue stiffness and promoting tumor expansion and migration. Interestingly, the common FGFR3 mutation decreases TGF-β within the TME, potentially leading to a less fibrous tumor and, therefore, a less aggressive phenotype [27,52]. This may explain why FGFR3 mutations are present in 70% of NMIBC cases but only 15% of MIBC [28].

### 2.4. Immune Landscape in NMIBC

#### 2.4.1. Immune Cell Infiltration

**Figure 2:**
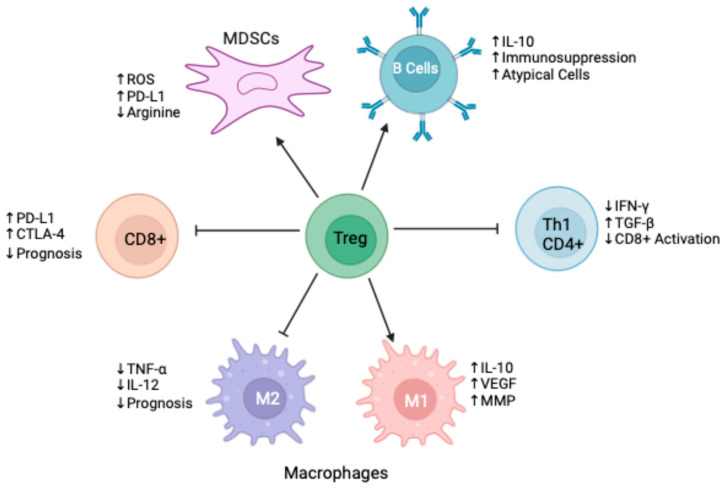
The role of Tregs within the NMIBC TME. Tregs secrete cytokines that interact multiple immune cells within the TME. Overabundance of Tregs can negatively regulate effector immune cells while stimulating tumorigenic cells such as MDSCs. Tregs may also modify the phenotypes of immune cells to make them tumorigenic.

The TME contains a diverse array of immune cells that influence the TME to either support or suppress tumor growth (Figure 2). This section will explore the relevant immune cell types within the TME o NMIBC.

CD8^+^ T cells are effector cells that recognize tumor-specific antigens, leading to a cell-mediated immune response. A higher density of CD8^+^ T cells within the TME is associated with a better prognosis in NMIBC [53]. Despite there being no morphological difference in CD8^+^ T cells in NMIBC [54], these cells often fail to infiltrate the tumor or are inactivated by immunosuppressive components of the TME. Higher-grade tumors are associated with higher concentrations of forkhead box protein-3 (FOXP3^+^) Treg cells, resulting in poor CD8^+^ T cell activation [55]. CD8^+^ T cells can also become exhausted in NMIBC, a phenomenon potentially attributed to high neo-antigen loads, leading to chronic CD8^+^ T cell activation and subsequent exhaustion [56]. It is crucial to note that the presence of CD8^+^ exhaustion results in the upregulation of PD-1 and CTLA-4, which may be a target for immunotherapies [57].

CD4^+^ T cells, or helper T cells, coordinate and amplify the humoral immune response. They recognize MHC class II molecules presented by DCs or macrophages and differentiate into various subsets, including T helper-1 (Th1), Th2, Th17, and Treg cells. Th1 cells secrete interferon-γ (IFN-γ), activating C T cells and promoting the expression of MHC molecules to boost antigen presenting capacity. Th2 cells inhibit IFN-γ and increase TGF-β, leading to a colder tumor environment [58]. BCG therapy in NMIBC has been associated with CD4^+^ T cell expansion, leading to anti-tumorigenic T cells whose subsets may serve as biomarkers for predicting BCG response [59,60]. Additionally, Treg cells, which promote a tumorigenic environment, show decreased concentrations in the TME following BCG therapy [61].

B cells, an understudied component of the NMIBC immune landscape, exert anti-tumor effects by producing tumor-specific antibodies and facilitating antigen presentation to T cells. In NMIBC, B cells can adopt a regulatory antigen phenotype by expressing IL-10 and other anti-inflammatory or immunosuppressive cytokines that dampen the T cell response [62]. BCG-resistant patients exhibit atypical B cells within tertiary lymphoid structures and promote immunosuppression in a mouse model [63]. Interestingly, females tend to promote more B cells within NMIBC, and this is associated with worse outcomes [64]. Activated B cells can also express PD-1, leading to immunosuppression [65].

TAMs, polarized to an M2 phenotype, promote tumor progression by releasing anti-inflammatory cytokines, stimulating angiogenesis, and remodeling the ECM. Infiltration of TAMs in NMIBC may be associated with increased angiogenesis, tumor grade, and worse prognosis [66,67]. Inhibiting TAM signaling in MIBC reduces cytokine secretion and enhances CD8^+^ T cell cytotoxicity by downregulating PD-1 [68]. The ratio of M1 to M2 macrophages has been suggested as a prognostic biomarker in bladder cancer, with a lower ratio associated with worse prognosis [69].

NK cells are innate lymphocytes capable of recognizing and killing cells without prior sensitization. In NMIBC xenograft mouse models, NK expansion is associated with potent cytotoxicity against bladder cancer cells [70]. BCG may upregulate the expression of CD56 and CD16 on NK cells, increasing their cytotoxicity [71]. Interestingly, NK cell counts have also been proposed as a potential biomarker for NMIBC recurrence; stromal NK counts are significantly higher in Ta tumors than controls, and patients with increased counts are significantly more likely to have recurrent NMIBC [72]. This paradoxical finding may suggest that while NK cells have the potential to exert anti-tumor effects, their functional activity rather than their mere presence plays a critical role in tumor control.

MDSCs are a heterogenous population of immunosuppressive cells that contribute to tumor progression through multiple mechanisms. The two main subtypes of MDSCS include monocytic (M-MDSCs) and granulocytic (PMN-MDSCs). These cells impair CD4^+^ and CD8^+^ T cells by inducing ROS, depleting arginine, and increasing PD-L1, B7, and FasL expression. These markers induce Tregs, M2 TAMs, and impaired DC function [73,74,75,76], creating a tumorigenic and immunosuppressive environment. MDSC recruitment is correlated with progression and prognosis in bladder cancer, likely due to significant inhibition of T cell proliferation [77,78,79].

Smoking is a leading risk factor for developing NMIBC. As previously mentioned, smoking contributes to 50% of diagnoses [8]. Smoking is typically associated with increased immunosuppression and can lead to altered immune cell and cytokine profiles, increasing IL-6, TNF-α, and C-reactive protein [80]. Paradoxically, smokers with NMIBC tend to have longer response duration to BCG therapy, which may be related to enhanced innate immune activation despite smoking-induced immune suppression, heightened Th1 responses, and BCG potentially overcoming immunosuppression. One study demonstrated that eosinophilia is associated with NMIBC recurrence; however, smoking typically lowers eosinophil counts. Since smokers also exhibit longer responses to BCG therapy, one hypothesis could be that smokers exhibit lower eosinophil levels, which may partly explore this response [81].

#### 2.4.2. Cytokine Milieu

Immune cells in the NMIBC TME produce many cytokines that influence the noncellular components and help determine the aggressiveness and therapy response of the cancer. Pro-tumorigenic cytokines include IL-6, TGF-β, vascular endothelial growth factor (VEGF), and IL-10, while anti-tumor cytokines include IFN-γ, tumor necrosis factor-α (TNF-α), and IL-12 [82].

IL-6 promotes tumor growth, immune modulation, and inflammation by activating STAT3 pathways, which maintain constitutive nuclear factor-κB (NF-κB) signaling. STAT-3 activation via IL-6 is associated with more aggressive tumor behavior, worse outcomes, shorter survival times, and more advanced NMIBC [83]. IL-6 overexpression in other cancers is typically associated with poor prognosis, although this relationship remains unstudied in NMIBC to our knowledge [84]. TGF-β mediates immunosuppression and tissue remodeling, promoting tumor proliferation, invasion, and epithelial to mesenchymal transition (EMT) by downregulating E-cadherin and upregulating matrix metalloproteinases (MMP) and vimentin genes [85]. Central to angiogenesis, VEGF enables tumors to form new blood vessels. VEGF overexpression in NMIBC is associated with shorter overall survival and recurrence predictions [86,87]. VEGF is produced by tumor cells, TAMs, and fibroblasts [88]. IL-10 is an immunosuppressive cytokine that inhibits the function of CD8^+^ T cells macrophages, APCs [89]. Urinary and serum IL-10 levels are biomarkers for tumor occurrence, while the IL-6 to IL-10 ratio may be a potent predictor of NMIBC recurrence [90,91].

Moving on to antitumorigenic cytokines involved in NMIBC, IFN-γ enhances antigen presentation and cytotoxic T-cell responses to combat tumor proliferation. Co-culturing BCG-resistant tumors with IFN-γ upregulates PD-L1 and HLA-E, showing potential for therapies combining anti-PD-L1 and BCG immunotherapies [92]. Repression of IFN-γ is more common in recurrent NMIBC, promoting a tumorigenic environment [93]. TNF-α plays a dual role within the TME. It promotes an inflammatory environment which stimulates immune cell recruitment to the tumor site [94]. However, chronic TNF-α stimulation can enhance tumor survival and proliferation through NF-κB activation, as well as metastasis through the induction of adhesion molecule expression, causing the negative effects of TNF-α to outweigh any benefit [95,96,97]. IL-12 bridges innate and adaptive immunity by promoting Th1 cell responses, activating NK and CTLA cells and strengthening anti-tumoral immunity [98]. A phase-1 clinical trial examining an oncolytic virus that increased IL-12 found significant tumor growth inhibition [99]. IL-8 promotes tumor growth by attracting neutrophils and macrophages to tumor regions. These cells promote angiogenesis and TME remodeling, which enhances tumor progression [100,101]. Elevated levels of IL-8 serve as biomarkers for NMIBC recurrence [100,101]. Finally, IL-17 is a pro-inflammatory cytokine produced by Th17 cells that contributes to bladder cancer occurrence and development by recruiting MDSCs and stimulating inflammatory cytokines [102]. The overexpression of IL-17 seen in bladder cancer is associated with higher recurrence rates [102].

#### 2.4.3. Immune Evasion Mechanisms

NMIBC employs a variety of immune evasion mechanisms to circumvent host immune surveillance, facilitating tumor survival and progression. These mechanisms include the downregulation of antigen presentation pathways and the upregulation of inhibitory immune checkpoints, inducing T cell exhaustion, the recruitment of M2 TAMs, and the secreting immunosuppressive factors [103,104].

NMIBC tumors frequently exhibit reduced expression of MHC class I molecules on tumor cells, impairing the ability of cytotoxic CD8^+^ T cells to recognize and destroy tumor cells. This is especially prevalent following BCG therapy. The study also noted that this was due to intracellular infection in cancer cells leading to downregulation of MHC class I molecules via autophagy [105]. Additionally, the inhibition of FGFR3 has been shown to upregulate MHC class I expression in bladder cancer, implying that FGFR3 downregulates this expression normally [106]. In other cancers, it has been proposed that mutations in β2-macroglobulin or epigenetic silencing of MHC-related genes are associated with loss of MHC class I expression [107,108]. To our knowledge, there are no papers that explore these mechanisms in NMIBC, but there may be potential mechanisms for this observation.

**Figure 3:**
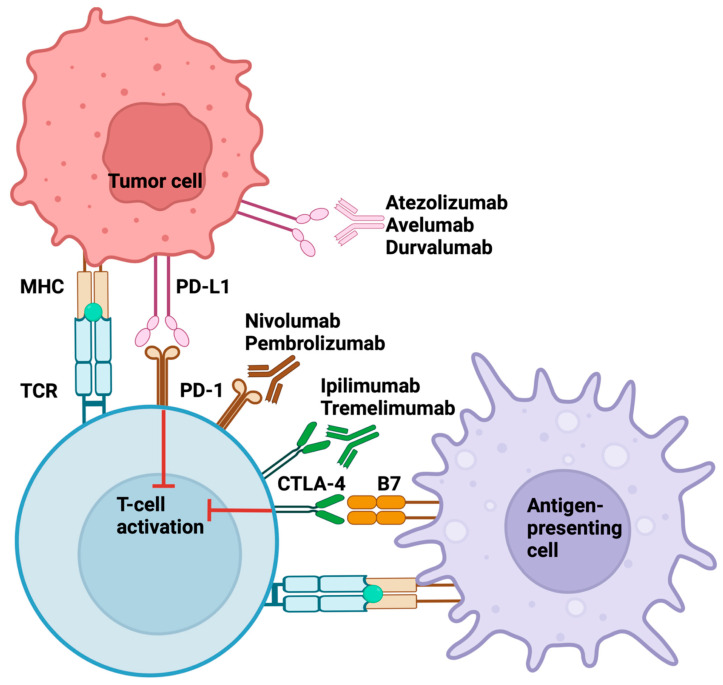
Interactions between PD-L1 and PD-1, CTLA-4 and B7, and the target sites of ICIs. PD-1 interact with PD-L1 leading to inhibition of T cell activation. Atezolizumab, avelumab, and durvalumab bind to PD-L1, while nivolumab and pembrolizumab target PD-1, preventing T cell activation. CTLA-4 binds to B7 leading to inhibition of T cell activation. Ipilimumab and tremelimumab bind to CTLA-4, preventing its recognition of B7 and subsequent inhibition of T cell activation.

Tumor cells and stromal cells within NMIBC overexpress immune checkpoint molecules such as PD-L1 and CTLA-4 (Figure 3). The upregulation of immune checkpoint molecules is performed to prevent autoimmunity in a normal immune response; however, cancer cells use this as an immunosuppression mechanism. Upon T cell activation, PD-1 is expressed on the T cell surface and can interact with PD-L1, which is commonly upregulated on the cancer cells. In BCG-resistant NMIBC tumors, an increased number of PD-L1 expressing cells may be a sign of immune escape [109]. Similarly, there may be upregulation of CTLA-4 on Tregs, which compete with CD28 over binding to DC8-/CD86 on APCs. This can subsequently deactivate the effector T cells [110]. Combining BCG and anti-CTLA-4 or anti-PD-1 therapies may enhance the immune activity within the NMIBC tumors and lead to a more efficacious immune response against the cancer [111]. This issue will be mentioned in future sections.

Chronic antigen stimulation within the TME can drive T cell exhaustion, which is associated with higher rates of recurrence after BCG therapy [104]. T cell exhaustion is characterized by sustained upregulation of inhibitory receptors such as PD-1, Tim3, CTLA-4, and LAG3 [112,113]. Exhausted T cells exhibit impaired cytotoxicity, limiting their capacity to mount an effective anti-tumor immune response, resulting in tumor escape [114].

TME results in the recruitment of a range of immunosuppressive cell populations, including Tregs, MDSCs, and TAMs, particularly with an M2 phenotype. These cells contribute to immune evasion in multiple manners, including immune checkpoint expression, cytokine production, and hypoxia. Tregs and TAMs will secrete cytokines such as TGF-β, IL-10, and PGE2 [115]. Additionally, MDSCs can cause arginine depletion within the TME, resulting in effector T cell dysfunctionality [116]. Finally, hypoxia may be found in higher-grade bladder tumors. The HIF-1α transcription factor is upregulated in some tumors, resulting in a more hypoxic tumor [116,117]. This may lead to lactate accumulation, causing the dysfunctionality of effector T cells.

## 3. PD-L1 in NMIBC

### 3.1. Biological Role of PD-L1

The PD-1 gene was discovered in 1992 by Ishida et al. and found to be associated with cell death as the mRNA is elevated upon the induction of programmed cell death and encodes a transmembrane protein but alone cannot induce cell death [118]. This led investigators to hypothesize PD-1 was involved in signal transduction and association with other proteins. In 1999, PD-L1 (B7-H1) was discovered by Dong et al. and found to cause secretion of IL-10, a cytokine that plays a role in activated T cell death in co-stimulated T cells in an IL-2 dependent manner [119,120,121]. These two studies were linked shortly thereafter by Freeman et al., who demonstrated that the ligand of PD-1 is PD-L1, and their interaction causes the inhibition of T cell receptor (TCR)-mediated proliferation and secretion of cytokines [122].

PD-1 has a cytoplasmic domain embedded in an immunoreceptor tyrosine-based inhibitory motif (ITIM), which is phosphorylated upon activation and recruits src homology 2-domain-containing tyrosine phosphatase 2 (SHP-2), which inhibits B cell receptor (BCR) signaling through the dephosphorylation of BCR signal transducers [123,124]. The cytoplasmic tail of PD-1 also contains an immunoreceptor tyrosine-based switch motif (ITSM), which recruits inhibitory SHP-1 and SHP-2 upon stimulation of T cells, but ligation with PD-L1 is necessary to inhibit T cell activation [125].

PD-L1 is upregulated on tumor cells in response to INF-γ, leading to an increase in IL-10 secretion and activated T cell death, making tumor cells more resistant to cell death [126]. In PD-L1 positive bladder cancers, tumor cells can evade the immune system through the downregulation of activated T cells, making this pathway a prominent target for ICIs and the development of new immunotherapies.

### 3.2. PD-L1 Expression in NMIBC

There is much discrepancy in the literature regarding PD-L1 expression in NMIBC and its prognostic significance. Nakanishi et al. found that PD-L1 overexpression was significantly associated with higher WHO grade tumors and higher recurrence rates [127]. These finding have been supported by several other studies finding association of PD-L1 with more aggressive cancer phenotypes, reduced rates of RFS, and overall significance as a prognostic biomarker [128,129,130,131,132]. This contrasts with other studies which have found increases in PD-L1 expression to be linked to better RFS and progression-free survival (PFS) [133,134,135,136]. Furthermore, other authors have found little to no correlation between PD-L1 and patient outcomes, suggesting no prognostic implications [109,137,138]. Statistically different levels of PD-L1 expression between tumor stages have been observed, with higher expression found in T1 and CIS tumors than low-grade Ta tumors [127,128,130,132,134]. This variable expression contributes to some of the discrepancies found regarding prognostic significance of PD-L1 in NMIBC, in combination with different assessment methodologies employed by these authors. Interestingly, a study also found PD-L1 expression to be higher in female patients with high-grade tumors than their male counterparts, which may contribute to shorter RFS and progression-free survival (PFS) rates seen in women, adding another variable to understanding the role of PD-L1 in NMIBC [64]. Careful evaluation is necessary when interpreting PD-L1 expression in NMIBC, and standardized assessment methods would be valuable in determining the prognostic significance of PD-L1.

The prognostic role of PD-L1 in BCG immunotherapy response has been the subject of more investigation. PD-L1 expression was reported to be highest within CIS tumors and granulomata of BCG-resistant patients by Inman et al., leading to the hypothesis that the inhibitory effect of PD-L1 on T cells was negatively impacting BCG therapy efficacy [139]. Over a decade later, Hashizume et al. reported that PD-L1 expression was significantly increased following BCG treatment in BCG-resistant NMIBC patients [140]. These findings suggested the predictive significance of PD-L1 in determining whether a patient would be responsive to BCG therapy. This hypothesis has been tested with variable conclusions; several studies have found no association between PD-L1 expression and BCG failure but affirmed increased expression of PD-L1 following BCG therapy [141,142]. Alternatively, Kates et al. found no difference in PD-L1 expression before and after BCG treatment and that PD-L1 positivity was predictive in determining BCG response, and Pierconti et al. found PD-L1 expression (using 22C3 assay) correlates with BCG failure [143,144]. The discrepancies found between these studies may result from different methodologies used included differing antibody clones in IHC analysis, varying tumor microenvironments causing studies to lack comparability, and small sample sizes [145]. Although PD-L1 has not been established as a prognostic biomarker for BCG response in NMIBC, it does appear to be upregulated in high-grade and/or BCG non-responsive tumors, suggesting that ICIs could be a useful therapy in conjunction with BCG.

### 3.3. Targeting PD-L1 in NMIBC

Avelumab, a human IgG1, PD-L1 inhibitor, was found to slow tumor growth and reduce tumor volume in mice with NMIBC [146]. In a rat model, Wang et al. found that anti-PD-L1 therapy, in combination with BCG, significantly decreases tumor weight, upregulates tumor infiltrating CD8^+^ T cells, causes them to produce significantly more granzyme B, INF-γ and TNF-α, and decreases myeloid-derived suppressor cells [147]. These promising results have led to further investigation of PD-L1 inhibitors in the treatment of high-risk and/or BCG resistant NMIBC.

A single-arm, phase II clinical trial assessed the efficacy of atezolizumab, a human monoclonal antibody (mAb) PD-L1 inhibitor, on high-risk, BCG-unresponsive NMIBC patients [148]. The complete response (CR) rate of the CIS group (*n* = 74, ±Ta/T1) was 27% at six months, the Ta/T1 group (*n* = 55) showed a 49% event-free survival (EFS) rate at 18 months, and the median duration of response (DOR) was 16.5 months. Following this study, a phase 1b/2 study investigating atezolizumab in high-risk, BCG-unresponsive NMIBC was conducted [148]. Patients were divided into two cohorts; those in 1A and 1B received 1200 mg of atezolizumab administered IV, while 1B also received six weekly doses of BCG with maintenance. The six-month CR rate was 33% in cohort 1A and 42% in cohort 1B. In 1A, the median DOR was 6.8 months, while in 1B the DOR was not reached but was ≥12 months. These data suggest that atezolizumab has clinical activity and is more effective in combination with BCG therapy.

Another IgG1 mAb targeting PD-L1 in clinical trials is durvalumab. A phase I trial involving 28 patients was conducted to investigate the effect of durvalumab alone, as well as in combination with BCG and external beam radiation therapy (EBRT) in NMIBC [149]. The three-month CR rates were 33% for durvalumab, 85% for durvalumab with BCG, and 50% for durvalumab with ERBT. A single-arm, phase II trial with high-risk, BCG-unresponsive, NMIBC patients (*n* = 17) found limited efficacy of durvalumab, with all but two patients discontinuing treatment due to disease persistence [150]. These studies indicate that durvalumab might be effective in combination therapies, particularly with BCG, but lacks efficacy as a monotherapy.

Cetrelimab, a human IgG4 mAb that binds to PD-1 [151], was studied individually and in combination with TAR-200, a continuous intravenous gemcitabine delivery system within the bladder, in SunRISe-1, a phase 2b trial investigating its effects on BCG-nonresponsive patients [152]. The CR rate was 68% for TAR-200 with cetrelimab, 84% for TAR-200 alone, and 46% for cetrelimab alone [153]. Due to the high efficacy of TAR-200, a phase III study is being conducted investigating its effects with and without cetrelimab, which showed unremarkable CR rates as a monotherapy in the previous trial [154].

Pembrolizumab became FDA-approved as an intravenous PD-1 inhibitor for high-risk, BCG-nonresponsive NMIBC patients with CIS after the KEYNOTE-057 single-arm, multicenter, phase 2 study [155]. Of the 96 patients in cohort A that received pembrolizumab, 41% had a CR at three months, 46% of these responses lasted 12+ months, and median CR time was 16.2 months. KEYNOTE-676 is an ongoing, phase III study focused on pembrolizumab and BCG combination therapy vs. BCG monotherapy efficacy (CR and EFS) in high-risk NMIBC [156,157].

A phase I trial of intravesical pembrolizumab and BCG combination therapy was conducted and had 6-month and 1-year RFS rates of 67% and 22%, respectively [158]. Additionally, this study found significant increases in CD4^+^ and CD8^+^ T cells following intravesical pembrolizumab treatment, a similar result to what was found in avelumab studies, as well as decreases in T cell exhaustion markers [158]. These findings indicate that pembrolizumab has the potential as both a combination and monotherapy to induce immune responses in high-risk NMIBC.

## 4. CTLA-4: Roles and Inhibition in NMIBC

### 4.1. Mechanisms of Action: Inhibition of Early T Cell Activation

Immune responses are necessary to recognize, remove, and remember pathogens that cause harm to the body. If the immune system is not turned off, this could lead to autoimmune conditions. Therefore, a mechanism is needed to turn off the immune system when necessary. One of these mechanisms is through CTLA-4, which is expressed on the surface of activated T lymphocytes and Tregs. Within a healthy individual, CTLA-4 molecules prevent autoimmune conditions by turning off the immune response, but certain conditions can hijack these mechanisms to promote their proliferation [159].

To fully activate, T lymphocytes need to bind to an APC and co-stimulatory molecules. CTLA-4 will compete with the CD28 co-stimulatory molecule for binding to B7 on the APCs. CTLA-4 binds with higher affinity than CD28; therefore, when overexpressed, CTLA-4 always outcompetes B7. Once CTLA-4 binds to B7, it will signal the T lymphocyte to dampen its immune function, leading to decreased T cell proliferation, cytokine production, and cytotoxic functions. In Treg cells, CTLA-4 will not dampen their function, instead, it will enhance Treg’s ability to suppress immune responses and immune tolerance [160].

In T lymphocytes, CTLA-4 binding to B7 molecules will result in intracellular signaling pathways. The cytoplasmic tail of CTLA-4 will contain two motifs: a tyrosine-based inhibitory motif (ITIM) and a tyrosine-based motif (ITSM). After activation of these motifs, the SH2 domain-containing protein tyrosine phosphatase 2 (SHP-2) and protein phosphatase 2A (PP2A) will be recruited. SHP-2 and PP2A dephosphorylation lead to important signaling molecules such as CD3 and ZAP-70 in the TCR signaling cascade. This will turn the TCR off by attenuating the activation signals (1560).

### 4.2. Impact on Tregs in the TME

As aforementioned, CTLA-4 plays a critical role in the function of Tregs. Mechanistically, it is proposed that CTLA-4 signaling in Tregs will remove B7 costimulatory molecules on the surface of APCs via trans-endocytosis. This will reduce the availability of co-stimulatory signals for the effector T cells, which will further inhibit their activation [161].

Within cancer, Tregs play a key role in shaping the TME by promoting an immunosuppressive environment that facilitates tumor progression. Tregs express FOXP3, CD25, and CTLA-4 in higher amounts [162]. Tregs tend to be upregulated following BCG therapy, which may promote resistance to this therapy [163]. Tregs will also further promote their immunosuppressive effects by secreting non-inflammatory cytokines such as IL-10 and TGF-β amongst other cytokines [164]. Tregs may also accumulate adenosine and deplete tryptophan via distinct pathways, which removes these key nutrients away from effector immune cells. Tryptophan depletion is caused by indoleamine 2,3-dioxygenase (IDO), an immunosuppressive enzyme that plays a critical role in creating a tolerogenic TME, and it is induced by Tregs. IDO will catalyze tryptophan breakdown, which is a key amino acid required for T cell proliferation and effector function into immunosuppressive metabolites such as kynurenine [164]. In a small study including 41 patients, IDO-positive bladder cancers were correlated with tumor progression. Further studies are necessary to explore the potential of IDO as a biomarker [165]. Adenosine accumulation occurs when CD39 and CD73 expressed on the surface of Tregs are upregulated. These markers act as ectonucleotidases that hydrolyze ATP down to adenosine. Adenosine then suppresses effector T cells [164]. More studies are necessary to uncover the potential role of IDO and adenosine in NMIBC, but it may be another dimension to consider when combatting an immunosuppressed TME.

The impact of Tregs on NMIBC is conflicted since high levels of Tregs may result in the suppression of the anti-tumor immune response [166]. Other studies have shown that Tregs may increase recurrence-free survival; this report did not state the effects on other T cell populations [167]. Further research is necessary to clear up the conflicting reports in the literature. It is well established that BCG therapy in NMIBC treatment will induce Treg cells within the TME [163]. Mechanistically, the demethylation of 11 signature genes is present in Tregs, including FOXP3, CTLA-4, Fas, and IL-2. These epigenetic modifications are associated with the long-term induction of Tregs [163]. High levels of Treg activation can limit the effectiveness of anti-tumor immune responses by upregulating PD-1, potentially setting the precedent for BCG and PD-L1/PD-1 blockade therapies [168]. Mechanistically, BCG therapy is an attenuated strain of *Mycobacterium bovis*, which is instilled into the bladder. The bacteria are internalized by urothelial cells and APCs, which leads to the production of pattern-associated molecular patterns (PAMPs). This triggers an innate immune response characterized by CD4^+^ T cell cytokine release, including IL-1, IL-2, IL-6, IL-10, IL-12, TNF-α, and IFN-γ, amongst others. This cytokine release causes the recruitment of other immune cells, including macrophages, neutrophils, and CD4^+^, CD8^+^ T cells [169]. However, the subsequent induction of Tregs suggests a complex immunoregulatory balance that may influence BCG efficacy.

### 4.3. CTLA-4 Expression in NMIBC

Tregs tend to be upregulated in more advanced NMIBC, older patients, and female patients. Additionally, TAM counts were significantly correlated with Treg count and IL-6 levels [170]. Tregs and CTLA-4 typically have a symbiotic relationship because Tregs commonly use CTLA-4 to affect immunosuppression, which leads to enhanced expression of CTLA-4 [171]. Anti-CTLA-4 antibodies in cancer therapeutics can bind CTLA-4 with high affinity and block the binding to CD80 and CD86. This causes enhanced antibody dependence cell cytotoxicity (ADCC) and depletion of Tregs in the TME, setting the precedence for further CTLA-4 ICI therapies [172]. CTLA-4 expression seems to be upregulated in serum for patients associated with higher grade NMIBC tumors, reflecting a more immunosuppressive environment [173,174]. Additionally, the presence of CTLA-4 and PD-L1/PD-1 are associated with an inflamed TME, but they are not associated with a higher risk of recurrence [174]. These studies provide a tumor profile for patients who may benefit from ICI therapies.

The expression of immune checkpoint molecules has been proposed as a biomarker for predicting ICI efficacy. Therefore, it would be important to compare PD-L1/PD-1 to CTLA-4 expression to select which ICI may be more effective. PD-L1 expression is upregulated following BCG therapy, potentially leading to BCG resistance [111]. This highlights the potential role of anti-PD-L1 ICI therapy following BCG therapy to help prevent resistance. Higher PD-L1 expression is often observed in high-grade and advanced-stage tumors, highlighting a potential patient profile that may respond better to anti-PD-L1 ICI [175]. PD-L1 expression has also been proposed as a prognostic biomarker that may be used to refine the risk stratification of NMIBC [128]. One study exploring this found that absolute levels of PD-L1 expression were predictive of disease-free survival; however, when comparing PD-L1 positive to PD-L1 negative patients, there was no significant difference in disease-free survival [142]. PD-L1 expression in patients seems to be relatively low with one study indicating 9.4% expression and another indicating 6.8% expression [138,176]. Interestingly, PD-L1 expression correlates with recurrence, with 11.6% showing high levels of PD-L1 expression [138].

Studies have indicated that CTLA-4 and PD-L1 can be co-expressed within the TME of NMIBC. This suggests that multiple immune checkpoints may be utilized by cancer to promote an immunosuppressive environment. The co-expression of these molecules is associated with more inflamed tumors but not with higher recurrence risks or prognostic value [174]. This presents clinical complications because the tumor can utilize multiple mechanisms to avoid immune detection. It also means that multiple immune checkpoints may need to be inhibited to overcome this co-expression [103,177]. There is, of course, tumor heterogeneity that exists, which makes it more complicated to focus on tumors that may be targeted by these immune checkpoints. Certain subtypes are more likely to express these immune checkpoints than others, and it should be considered during treatment [178]. Further research is necessary to elucidate the cancer profiles that respond best to immunotherapies in NMIBC.

CTLA-4 inhibitors in NMIBC are still under investigation, with little research published on this topic compared to PD-L1 inhibitors. Similarly, PD-L1 inhibitors are less studied clinically. There have been metastatic clinical trials, in addition to the few NMIBC trials discussed previously, that have investigated PD-1/PD-L1 inhibitors, such as CheckMate-901, Keynote-361, and IMvigor130. CheckMate-901 and IMvigor130 found that overall survival increased with PD-1/PD-L1 combined with traditional chemotherapy, while Keynote-361 failed to meet their end goal [179,180,181]. The relative success of PD-L1/PD-1 inhibitors set the precedent for further exploration of CTLA-4 inhibitors in the hope of finding a combination therapy in UBC.

The upregulation of CTLA-4 and PD-1/PD-L1 does not always predict immune blockade success and should not be used as the only predictor of success for ICI efficacy [182]. Other biomarkers are necessary to predict efficacy. This depends on tumor type, microsatellite instability, neo-antigen load, and immune infiltration [183]. Each of these factors can vary significantly between patients and tumor types; this highlights the importance of creating a tumor profile and then using data from that profile to predict treatment efficacy.

### 4.4. Targeting CTLA-4 in NMIBC

CTLA-4 ICI Ipilimumab (Yervoy) has been approved by the FDA for multiple cancers, including melanoma, renal cell carcinoma, and non-small cell lung cancer. These blockades have shown efficacy in other cancers, including mesothelioma, head and neck cancers, breast cancer, and ovarian cancer, amongst others [184]. Early studies on NMIBC have shown that CLTA-4 inhibitors may be efficacious in tumor models; however, there have been limited clinical trials that explore this. One study found that increases in inducible costimulator (ICOS) have a dual role in autoimmunity and immunosuppression; this pathway resulted in a better anti-CTLA-4 response in bladder cancer patients [160,185]. Additionally, one study by Sharma et al. found that anti-CTLA-4 immunotherapy exerts its effects by increasing effector T cells but does not deplete Tregs within the TME [186]. This result was mirrored in another study by Zhang et al., that found that CTLA-4 disrupted cytotoxic lymphocytes (CTLs) in bladder cancer in xenograft mouse models. CTLA-4 inhibitors were more successful in MIBC than in NMIBC [187]. Combinations of anti-CTLA-4 inhibitors and PD-1/PD-L1 inhibitors may be better than both treatments in monotherapy. A combination of this therapy has been shown to decrease IL-6 serum levels and resulted in tumor regression in xenograft mouse models. One concern of this combination therapy is the increased toxicity that accompanies it; this may put emphasis on exploring less toxic immune checkpoint combinations such as anti-PD-1/anti-LAG-3 [188]. While more studies are necessary to solidify our understanding of anti-CTLA-4 therapy in bladder cancer, there is promising evidence that these could make a difference in treating NMIBC.

A key phase 3 clinical trial in 2020, DANUBE, had three treatments, including durvalumab (PD-L1 inhibitor), tremelimumab (CTLA-4 inhibitor), and chemotherapy. They then divided the patients into three groups: cisplatin eligible, PD-L1 status, and presence of metastases. They found that in the high PD-L1 group, patients receiving durvalumab had a longer overall survival. Additionally, patients in an intent-to-treat group’s mean overall survival for patients receiving durvalumab and tremelimumab was 15.1 months, compared to 12.1 months in the chemotherapy group. These results were not statistically significant but showed numerical difference. The adverse events found in this trial were significantly decreased in the immunotherapy groups, indicating that it may decrease negative side effects [189]. While these results were not statistically significant, more clinical research should go into deciphering patterns in patients where immunotherapy is successful. Additionally, the apparent drop in adverse outcomes and side effects may lead to better patient tolerance for cancer therapy.

While the large DANUBE clinical trial did not come to an endpoint, the smaller NABUCCO trial examined 24 patients with more advanced bladder cancer. They treated patients with a combination of nivolumab and ipilimumab and found that the combined blockage of CTLA-4 and PD-1 was effective. 58% of patients in this trial had no remaining invasive disease, and 46% had a complete pathological response. They found that this was independent of CD8^+^ T effector cells, and there was an establishment of tertiary lymphoid centers [190]. This sets the precedent for further exploration into combination therapies, including anti-CTLA-4 drugs.

There have been a few studies exploring the role of PD-1/PD-L1 ICIs in different clinical situations, such as CheckMate-901, Keynote-361, and IMvigor130. There has not been significant exploration of anti-CTLA-4 inhibitors in bladder cancer. Additionally, these clinical trials specifically apply to MIBC; therefore, applying these results to NMIBC should be carried out with caution.

## 5. Conclusions

The roles of PD-L1 and CTLA-4 present compelling targets for therapy of NMIBC. PD-L1 expression has been associated with variable prognostic implications, with studies reporting both positive and negative correlations with RFS and PFS. While more research is necessary to elucidate its precise predictive value, its upregulation in BCG-nonresponsive tumors supports the rationale for PD-L1 inhibitors. Clinical trials have demonstrated that PD-L1 inhibitors, such as atezolizumab and pembrolizumab, show promise, in combination with BCG, in enhancing anti-tumor immune responses. Further clinical trials should investigate the use of these therapeutics in different conditions to pinpoint their efficacy.

Similarly, CTLA-4 tends to be upregulated in NMIBC and plays a role in the immunosuppressive environment through its interactions with Tregs. Modulation of the TME by Tregs promotes T cell interaction with CTLA-4 molecules, resulting in decreased cytotoxicity. This sets the rational for utilizing anti-CTLA-4 therapies against NMIBC. Although some studies support the efficacy of CTLA-4 inhibition, there is not enough literature published to fully endorse the clinical use of these therapeutics. We hope that more studies on this topic will be conducted in the coming years.

Despite advancements, challenges remain in standardizing biomarker assessment methodologies and optimizing patient selection for checkpoint inhibitor therapies. ICIs in other cancers show efficacy in subsets of patients but are unresponsive in other patients. Moving forward, further research should focus on identifying predictive biomarkers for ICI success to refine combination strategies. Ideally, clinicians would be able to profile their patients’ tumors and suggest effective treatments based on those biomarkers to address the unique characteristics of each patient’s tumor. Additionally, further understanding of the role of immunoregulatory molecules and mechanisms may enhance our understanding of these immune pathways and enhance therapeutic efficacy.
